# Dark Carbon Fixation: An Important Process in Lake Sediments

**DOI:** 10.1371/journal.pone.0065813

**Published:** 2013-06-11

**Authors:** Ana Lúcia Santoro, David Bastviken, Cristian Gudasz, Lars Tranvik, Alex Enrich-Prast

**Affiliations:** 1 Department of Ecology, Institute of Biology, University Federal of Rio de Janeiro, Rio de Janeiro, Brazil; 2 Department of Thematic Studies – Water and Environmental Studies, Linköping University, Linköping, Sweden; 3 Department of Ecology and Evolution - Limnology, Uppsala University, Uppsala, Sweden; National Institute of Water & Atmospheric Research, New Zealand

## Abstract

Close to redox boundaries, dark carbon fixation by chemoautotrophic bacteria may be a large contributor to overall carbon fixation. Still, little is known about the relative importance of this process in lake systems, in spite the potentially high chemoautotrophic potential of lake sediments. We compared rates of dark carbon fixation, bacterial production and oxygen consumption in sediments from four Swedish boreal and seven tropical Brazilian lakes. Rates were highly variable and dark carbon fixation amounted up to 80% of the total heterotrophic bacterial production. The results indicate that non-photosynthetic carbon fixation can represent a substantial contribution to bacterial biomass production, especially in sediments with low organic matter content.

## Introduction

The incorporation of inorganic carbon into organic matter, i.e. carbon fixation, is one of the essential functions in ecosystems. In addition to photosynthetic incorporation, chemoautotrophic organisms incorporate inorganic carbon to varying extents [1], [2], representing an autochthonous source of organic matter [3]. In deep ocean regions, chemosynthesis, and not the photosynthesis, is admittedly the main reduced carbon source [4], with chemosynthetic bacteria recognized as base of these ecosystems food webs [5]. The importance of chemosynthesis to trophic webs has been also reported to freshwater ecosystems by recent studies, pointing C-1 compounds (especially methane) as important carbon and energy vectors [6], [7].

Chemoautotrophic microbes require the simultaneous presence of oxidized and reduced compounds that will react and liberate energy needed to support inorganic carbon fixation in the absence of light (dark carbon fixation; DCF). Therefore, the most extensive rates of DCF are expected at interfaces between aerobic and anaerobic zones [8]. According to Detmer et al. [9], DCF at a marine pelagic redoxcline can reach up to 30% of the surface primary production. High DCF rates have also been reported at other marine redoxclines [10], [11]. From the 90′s on, chemosynthetic activity has been reported in fresh water ecosystems: Lake Cadagno (Switzerland), Lake Ciso (Spain), Lake Mekkojarvi (Finland), Lake Kinneret (Israel) among others [12]–[15]. In lake water columns, DCF has been reported to contribute from 0.3 to approximately 50% of the total CO_2_ fixation on a whole lake basis, with contributions >10% primarily in sulfide rich lakes [16].

Sediment-water interfaces of lakes and coastal environments offer sites of intense organic matter deposition and degradation [17], [18]. Biogeochemical activity is high and oxygen can be depleted in the upper few millimeters of the sediments, creating a steep chemical gradient that provides a microenvironment with high chemoautotrophic potential [19]. Processes of ammonium, sulfur and methane oxidation are examples of some redox reactions associated with chemosynthesis in lake sediments [16]. Some chemosynthetic processes can reach rates up to three orders of magnitude higher in the sediment than in the water column [20], and recent studies highlights the idea that chemosynthetic production could represent a paradigm shift in how we view production in fresh water ecosystems [21].

Recent literature emphasizes the relative importance of specific sediment chemosynthetic processes as energy sources for the food web [6], [22]–[25], [7]. Some studies suggest that the carbon flux from chemosynthetic activity may be more important in promoting lake food webs than expected. However, the quantitative role of bacterial chemoautotrophic activity and the importance of DCF in relation to sediment C cycling lake sediments are still unknown. The classic sediment-water interface carbon cycling considers the microbial oxidation of organic matter and the incorporation of dissolved organic carbon (DOC) into bacterial biomass via secondary production [26]. If sediment DCF is extensive it may be an important, but currently underestimated, source of biomass carbon to benthic food webs. One way to address the relative importance of DCF is to compare the rates with other metabolic processes in the sediment.

Temperature and organic matter quality and origin are important drivers of sediment bacterial metabolism and biomass [27], [28]. Bacterial metabolism in boreal lake sediments is constrained by low temperatures and by the recalcitrant nature of the dominant organic carbon, resulting in sediments being an effective sink of organic carbon [29]. Yet, tropical lakes, like most other lakes, show frequent CO_2_ supersaturation [30] and support intense metabolism [31], enhanced by their warm temperatures [32] and the high production of the tropical forests and grasslands in their watersheds [33].

In this study we compared sediment DCF, bacterial production (BP), and oxygen consumption (SOC) rates from boreal lakes in Sweden and tropical lakes in Brazil. We demonstrate that DCF rates can be relatively high and can represent an important contribution to bacterial biomass production, especially in sediments with low organic matter content. Tropical lake sediments have a much higher variability in all measured processes and present lower DCF and higher BP and SOC rates than boreal sediments.

## Materials and Methods

### Study site and sampling

The research was carried out from sediment sampled in four Swedish boreal lakes in May 2008 and seven Brazilian tropical lakes in September 2008. Surface water characteristics are presented in Table 1. Two of the Swedish lakes (Lötsjön and Fälaren) were thermally stratified during summer. All of the Brazilian lakes had a shallow non-stratified water column. The coastal tropical lakes are parallel to the shoreline and influenced by marine groundwater inflows. The other tropical lakes are located in the Paraguay River floodplain, one of the main rivers of the Brazilian Pantanal. Sediment cores were collected with plexiglass tubes in the littoral or sub-littoral zones at depths of 1–3 m. The bottom water at those locations was always oxic. No algae colonization was observed on the top of the sediment from the studied lakes. The water column from all lakes was colored, indicating the presence of high amounts of humic compounds, which decrease sunlight penetration. Lake bottom water was sampled in plastic carboys for later use during incubations. The sediment cores from each lake were transported to the laboratory with care to avoid re-suspension and to keep the sediment structure intact.

**Table 1:** Coordinates, sample site depth, water salinity, and sediment contents (% of dry weight) of water, sediment organic carbon and nitrogen content, and C:N ratio at the studied lakes.

	Lake	Coordinates	Depth (m)	Salinity (‰)	Water (%)	Org C(%DW)	N (%DW)	C:N
**Boreal**	Lötsjön	66^o^41′ N 16^o^20′ E	3.0	0.0	63.3	3.25^*±^2.91	0.19±0.10	16.69±6.21
	Långsjön	66^o^43′ N 16^o^20′ E	2.0	0.0	75.9	3.12±1.24	0.36±0.14	8.49±0.21
	Strandsjön	66^o^40′ N 15^o^76′ E	2.0	0.0	91.3	12.93±0.11	1.46±0.00	8.81±0.08
	Fälaren	66^o^92′ N 16^o^10′ E	1.3	0.0	93.0	22.98±1.10	1.93±0.10	11.86±0.23
**Coastal Tropical**	Carapebus	22^o^15′ S 41^o^35′ W	2.0	7.9	15.0	0.24±0.10	0.01±0.00	18.46±5.51
	Visgueiro	22^o^11′ S 41^o^24′ W	1.8	24.5	25.0	8.30±0.33	0.85±0.02	9.74±0.14
	Pires	22^o^10′ S 41^o^22′ W	1.8	29.7	27.1	9.38±0.43	1.14±0.01	8.24±0.48
**Tropical**	Teresa	18^o^57′ S 57^o^26′ W	2.0	0.0	59.7	1.34±0.08	0.10±0.03	15.23±6.59
	Presa	18^o^59′ S 57^o^25′ W	2.8	0.0	32.0	1.87±0.03	0.24±0.08	9.24±0.78
	Lobo	18^o^57′ S 57^o^36′ W	3.0	0.0	74.8	4.12±1.51	0.64±0.08	6.29±1.58
	L1	19^o^02′ S 57^o^55′ W	2.5	0.0	35.7	10.19±1.86	0.99±0.07	10.38±0.84

Numbers represent average and SD (n = 5).

### Sediment incubations

Sediment cores (4 replicates, approximately 7.0 cm of sediment and 6.0 cm of overlying water) were placed in small buckets with corresponding lake water from each site within around an hour of sample collection. A magnetic stirrer placed between the cores and magnet rods suspended in each core ensured gentle mixing and allowed water exchange between the cores and the surrounding water in the bucket. The whole setup was kept aerated using an aquarium pump outside the cores at 10°C for boreal and 25°C for tropical lakes during 5 hours for stabilization. Based on previous SOC and O_2_ profile tests, the time of 5 hours was more than enough to ensure sediment stabilization, avoiding artifacts in terms of e.g. O_2_ demand. After a first stabilization period, sediment oxygen consumption (SOC) was measured in whole-core incubations with start-end sediment-water interface incubation, according to Dalsgaard et al. [34]. SOC indicated total organic matter degradation and was a proxy for the mineralization rates in the studied sediments.

After a second period of stabilization the sediment cores were sliced and the uppermost centimetre was used, since the oxic-anoxic transition zone is usually located in this layer [35]. After sampling, the sediment was well mixed forming a slurry for DCF and BP measurements. Bacterial production was determined via ^3^H-leucine incorporation using sediment diluted 10 fold with lake water, in order to minimize the physical quenching. For this, 1 ml of upper centimetre sediment was mixed with 9 ml of 0.2 µm filtered lake water taken from above the sediment. 0.1 ml of the slurry was added in four 2 ml polypropylene tubes with screw caps: 1 blank (with 1.5 ml of borax buffered formaldehyde 4%) and 3 sample replicates, which received 50 µl of diluted isotope (158 Ci mmol^−1^ and 73 Ci mmol^−1^ for Swedish and Brazilian incubations respectively) to reach 250 µM final leucine concentration. All tubes were incubated in the dark. The incubation was stopped 2 to 3 hours later by borax buffered formaldehyde addition (to 4% final concentration). After that, the incubated samples were centrifuged (at 14000–16000 rpm for 10 minutes) and the supernatant was discarded. 1.5 ml of 5% trichloroacetic acid (TCA) was added to the sediment followed by intensive mixing by a vortex to wash the sediment. Thereafter the sediment was centrifuged again and the TCA solution was removed. This washing cycle was repeated two more times with 5% TCA and finally with 80% ethanol. After removing the ethanol, scintillation cocktail was added and incorporation of ^3^H into bacterial proteins was measured by liquid scintillation analysis. The conversion factor of leucine incorporation to bacterial carbon production (BCP) was based on the assumptions from Buesing and Marxsen [36], using the equation: BCP (Kg) = 1.44×Leu_inc_ (Leu_inc_ = leucine incorporation in mol).

Sediment dark carbon fixation (also known as bicarbonate fixation or chemosynthesis) was determined by incorporation of ^14^C labelled inorganic carbon (DI^14^C; added as NaH^14^CO_3_) using sediment slurry diluted 5 fold. The dilution was made with 0.2 µm filtered water taken from above the sediment. 1.5 ml of slurry was transferred to eight 20 ml scintillation vials per lake: 3 blanks and 5 sample replicates. In the Brazil incubations, 0.25 ml of borax-buffered 37% formaldehyde was added to the blanks prior to sediment slurry addition to immediately stop microbial activity. Each vial received DI^14^C (0.8 µCi in 0.4 ml aqueous solution, 58 mCi mmol^−1^). In the incubations of Swedish sediments microbial activity in the blanks was stopped by the addition of 0.5 ml 0.1 M HCl and 12.5 µCi of DI^14^C in 0.05 ml aqueous solution (specific activity 60 mCi mmol^−1^) was added to each vial. After a dark incubation period of 3 to 4 hours, microbial activity in sample replicates was stopped as in the blank vials. After that, 2 ml of 0.1 M HCl was added to each vial to convert DIC to CO_2_, followed by purging with compressed air for approximately 3 hours to remove remaining DI^14^C. This final slurry was diluted with pure water up to a volume of 10 ml and another 10 ml of InstaGel scintillation cocktail (Perkin Elmer) was added. The detected radiation in the blanks was subtracted from the radiation in the samples and this blank-corrected incorporation of ^14^C was used to calculate the fraction of added radioactivity that was fixed. The fraction was assumed to represent the fraction of the overall dissolved inorganic carbon (DIC) that was fixed. The dark carbon fixation was calculated by multiplying this fraction with the DIC concentration measured in the water just above the sediment (see below).

A separate test with sediment from the same core incubated using both Brazilian and Swedish protocols confirmed that the differences in terms of how microbial activity was stopped or how much isotope was added did not affect the results of bacterial production or dark carbon fixation assays. Another test with simultaneous slurry and stratified core incubations to DCF and BP measurements in the first centimeter of the sediment showed no significant difference between both incubation procedures (Unpaired t test, p<0,05, Santoro et al, conditionally accepted by Limnology and Oceanography Methods).

The same sediment used for the slurry incubation was subsampled and temperature air dried. Subsequently, the sediment was analyzed for the total C and N in an elemental analyzer (NA 1500, Carlo Erba instruments). The amount of dissolved inorganic carbon overlying water was determined with a Sievers 900 TOC analyser for the boreal lakes, or manually by sample acidification to pH below 2, static headspace extraction and gas chromatographic analysis of the extracted CO_2_ for the tropical lakes. The partitioning of CO_2_ between the headspace and the water was accounted for using Henry’s Law.

All necessary permits were obtained for the described field studies for Brazilian Environments. The permission for sampling at the National Park of the Restinga de Jurubatiba was issued by the Brazilian Institute Chico Mendes. There was not need to ask for permission to sample at the other tropical environments as they were not privately-owned or protected in any way. No specific permits were required for the described field studies in Sweden. The field studies did not involve endangered or protected species.

## Results and Discussion

**Figure 1:**
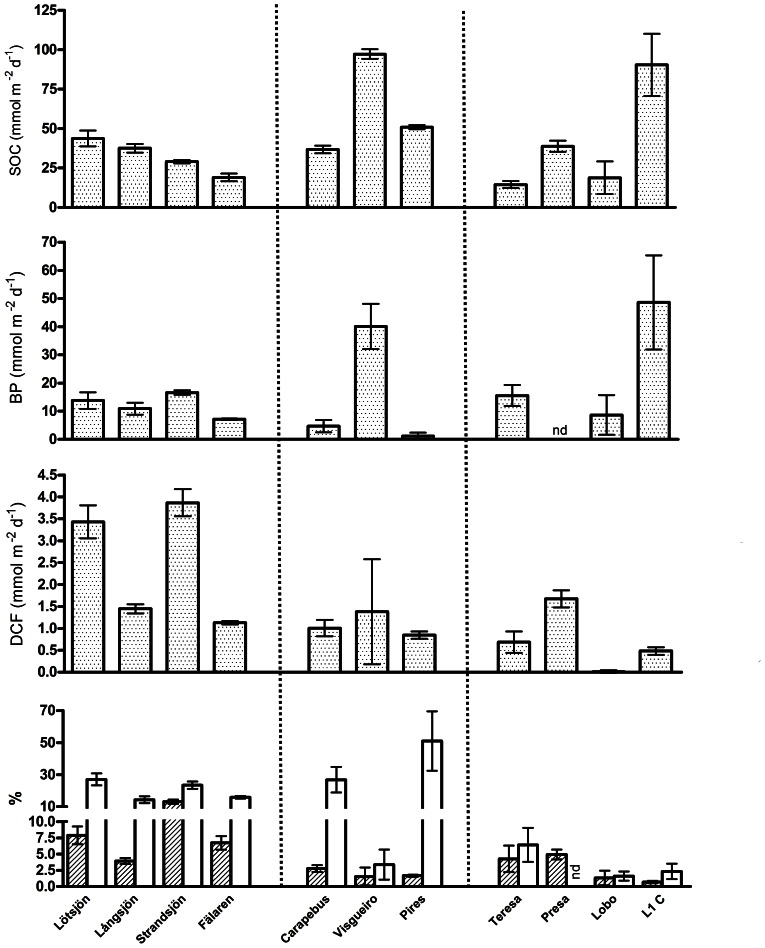
Comparison between measured sediment rates. Rates of sediment O_2_ consumption (SOC), bacterial production (BP), dark carbon fixation (DCF) and carbon fixation expressed as a percentage of sediment O_2_ consumption (hatched bars, DCF:SOC) and of bacterial production (white bars, DCF:BP) in the surface sediment for all studied lakes. Symbols represent average and plus-minus one standard error.

The measured rates were highly variable among the studied systems (Fig. 1). Some of the environments presented average rates of SOC higher than previously observed in a temperate eutrophic lake (27 mmol O_2_ m^−2^ d^−1^) or temperate reservoirs (45 mmol O_2_ m^−2^ d^−1^) [37], [38]. SOC average rates varied from 14.5 mmol O_2_ m^−2^ d^−1^ in Teresa to 97.3 mmol O_2_ m^−2^ d^−1^ in Visgueiro lake. Even though boreal sediments showed larger variation of organic carbon (OC) content (Table 1), SOC was more variable in tropical sediments than in temperate ones. As showed by Tomaszek & Czerwieniec [38], temperature can constrain SOC rates more strongly than organic matter concentration. Two tropical environments, Pires and L1, presented the lowest (1.2 mmol C m^−2^ d^−1^) and the highest (48.6 mmol C m^−2^ d^−1^) rates of sediment bacterial production (BP), and those values are also high compared to previous studies [39].

The highest rate of dark carbon fixation (DCF) was found in the boreal lake Strandsjön (4.0 mmol C m^−2^ d^−1^) and the lowest rate in the tropical lake Lobo (0.01 mmol C m^−2^ d^−1^). Our DCF estimates are high when compared with non-photosynthetic carbon fixation rates from marine sediments. Evrard et al. [40], showed a DCF rate of 1.98 µmol C m^−2^ h^−1^ in marine sediments, and according with the authors, mostly caused by phototrophs with only a small contribution by chemoautotrophic bacteria. This value is even lower than the observed in the tropical lake Lobo, which had the lowest DCF rates of the present study, and around three orders of magnitude below the rates found in lake Strandsjön. Despite dark carbon fixation (DCF) rates were generally higher in boreal sediments, our data can suggest that lake sediment chemoautotrophy is extensive both in boreal and tropical environments, and higher than previously demonstrated for marine sediments [40], [41]. Sediments from lakes and wetlands usually have higher microbial activities than marine ecosystems [42]. However, studies of dark carbon fixation in freshwater sediments are still few, and most of them evaluate specific chemosynthetic processes instead of DCF as a whole [43], [20].

The current data shortage on sediment DCF may be related to methodological limitations. The use of radioactive or stable isotopes makes the measurement by slurry incubations the most parsimonious way in most of the cases. Besides to be an easier approach, slurry incubations allow sediment dilution, which minimize the amount of radioactive material needed and the physical quenching, when the sediment itself acts as a physical barrier that decreases the fluorescence intensity of the isotope upon scintillation counting. Slurry incubations, as done in this study, may bias rates relative to field conditions, concerning DCF processes due the disruption of redox gradients [16]. However, shallow lake sediments, as the studied here, are usually disturbed by wind action [44] and bioturbation [45], [46], and therefore mixing similar to the slurry of the uppermost sediment applied in our experiments may not be uncommon in the upper centimeter sediment beneath shallow waters under *in situ* conditions. DCF and bacterial production rates from the first centimeter of Strandsjön lake were measured simultaneously in slurry and intact sediment incubations (Santoro, et al, Limnology and Oceanography Methods, conditionally accepted). The average rates using both procedures were very similar (1.54 mmol C m^−2^ d^−1^ and 1.48 mmol C m^−2^ d^−1^ for slurry and intact sediment respectively), with no significant difference between them (unpaired t test, p>0,05).

Given the relatively high presence of reduced and oxidized compounds in the surface of freshwater sediments, especially compared to the water column, and that surface shallow sediments experience turbulent mixing providing simultaneous access to such compounds, it should not be surprising to observe high rates of sediment DCF in many lacustrine systems. The aerobic slurry incubations may also be regarded as conservative estimates since chemosynthetic organisms can also be strictly anaerobic [16]. In addition, DCF was measured with the diluted sediment slurries, and dissolved inorganic carbon (DIC) concentration of the water above the sediment was used to calculate the rates. The water DIC concentrations are normally considered lower compared to the DIC concentrations found in the sediment pore water [47]. It is therefore likely that DCF rates presented in this study are conservative estimates. Notably, our results reflects exclusively the incorporation of dissolved inorganic carbon as CO_2_, and not chemosynthesis based on other C1 compounds, an important pathway of these process, in particular considering methanotrophic bacteria [6], [25].

The ratio sediment DCF:BP demonstrated that DCF corresponded from 8.41% to 37.4% of the total heterotrophic bacterial production in the studied boreal sediments. This chemoautotrophic contribution was very variable in tropical lakes, varying from 0.40% to 80.4%. A recent study showed that inorganic carbon fixation rates in surface deep-sea sediments accounted, on average, for 19% of the total heterotrophic biomass production [48], i.e. within the range presented here. DCF corresponded to between 3.03% and 15.93% of the SOC in boreal lakes, while this contribution varied from 0.14% to 9.38% in tropical systems (Figure 1).

As can be observed in Table 1, sediment organic carbon content varied up to two orders of magnitude between lakes, and two of the boreal environments had the highest values (Strandsjön and Fälaren). Despite that, boreal sediments showed lower rates of BP and SOC (Figure 1). Gudasz et al. [29] showed that, due the recalcitrant nature of the dominant organic carbon source of boreal lakes, the increasing organic carbon influence does not necessarily promote and enhancement in sediment bacterial metabolism.

**Figure 2:**
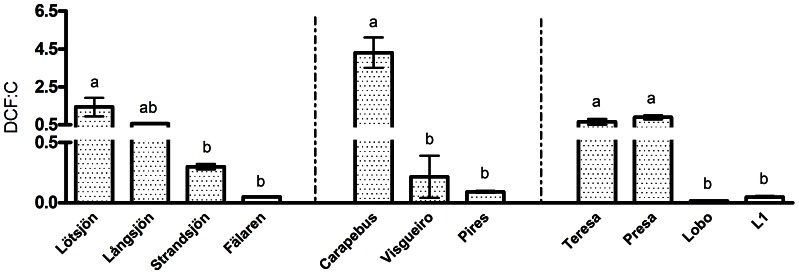
Ratio between dark carbon fixation (DCF; mmol C m^−2^ d^−1^) and organic carbon content (C; % of dry weight). DCF:C for studied boreal, coastal tropical and tropical group of lake sediments, respectively. Symbols represent means and bars show SEM (n = 5). Different letters indicate significant differences (ANOVA, p<0.05) between lakes within each group. Note different scales.

No relationship between DCF and SOC rates was found when considering all studied lakes (data not shown). We also did not found any direct correlation between DCF and the examined variables, as water salinity, pH or sediment contents of water, organic carbon and nitrogen (Spearman correlation, p>0,05). Understanding DCF regulatory factors and its relationship with environmental variables is an important aim, but it will demand a much larger dataset to achieve this. The DCF rates and the sediment OC content were used to construct a ratio that can be considered as an index that allows addressing the efficiency of the DCF activity per unit of OC content in each sediment. Sediments with lower OC content showed significantly higher values of DCF: OC content ratios (ANOVA, p<0.05) (Figure 2). No tendency was observed using data from all studied lakes, but there were apparent separate relationships for different kinds of lakes (i.e. boreal vs. coastal vs. tropical lakes), probably due different available substrates and dominant chemosynthetic processes occurring in each region. The lower DCF:OC ratio observed in sediments with higher carbon content may be caused by a competitive pressure imposed by heterotrophic community in these systems. This argument is also consistent with the positive correlation (Pearson, p<0.05) between BP and OC content observed in all studied systems with the exception of two lakes, Fälaren and Pires, where OC content was high and BP low. In general, the growth rates of chemosynthetic microorganisms are lower than for heterotrophic microorganisms, due to a lower energy yield of the associated redox reactions [16]. Consequently, high sediment organic carbon content can support a higher heterotrophic activity, able to suppress chemosynthetic processes by using oxygen, nitrate and other substrates more efficiently.

The DCF activity observed here suggests that the ability to perform inorganic carbon fixation is widespread in lake sediments. It has also to be taken into consideration that in addition to uptake by chemoautotrophic bacteria, heterotrophic bacteria can incorporate dissolved inorganic carbon via anaplerotic reactions (e.g. [2]). Anaplerotic CO_2_ fixation may account for some of the inorganic carbon uptake measured in lake sediments. The activity of the citric acid cycle requires anaplerotic reactions, where CO_2_ is incorporated, to restore the cycle intermediates that are withdrawn for the biosynthesis of cell constituents [49]. The most recently discovered anaplerotic reaction sequence forms malate and succinyl-CoA from three acetyl-CoA, one CO_2_ and one HCO_3_ in a linear pathway [50], and according to Schink [51], this new pathway is probably widespread among many metabolic groups of bacteria.

The uptake of inorganic carbon into heterotrophic bacteria is enhanced during slow growth and starvation [52]. Considering the slower community-wide specific growth of heterotrophic bacteria in sediments compared to the water column of lakes [53], a considerable fraction of the bacteria possess very slow growth, and possibly enhanced anaplerotic inorganic carbon uptake. This is in accordance with the tendency of higher DCF:OC ratios in sediments with lower organic carbon content, possibly causing less favorable conditions and hence slower growth. A similar argument of high dark carbon fixation in slow-growing or starving bacteria has recently been made with reference to pelagic bacteria in the Arctic Ocean during winter conditions [54].

This study shows that dark carbon fixation can be a quantitatively important carbon fixation pathway in sediments of inland water ecosystems. The results showed here draws the attention to chemosynthetic sources of carbon and energy, often disregarded so far, encouraging further studies especially in lake sediments. We observed highly variable rates of DCF between different studied lakes, indicating influence of different processes and regulating factors. Dark carbon fixation amounted up to 80% of the total heterotrophic bacterial production and we also showed a tendency of higher potential for DCF in boreal lake sediments. The present study goes against recent studies that neglect the importance of the chemosynthetic carbon fixation pathway (i.e. [55]). Chemosynthetic carbon fixation can challenge the traditional view that authochthonous carbon in lakes are produced solely by phototrophic carbon fixation.

## References

[1] Dijkhuizen L, Harder W (1984) Current Views on the Regulation of Autotrophic Carbon-Dioxide Fixation Via the Calvin Cycle in Bacteria. J Microbiol 50: 473–487. 10.1007/BF02386221 6099093

[2] Feisthauer S, Wick LY, Kastner M, Kaschabek SR, Schlomann M, et al (2008) Differences of heterotrophic (CO_2_)-C-13 assimilation by *Pseudomonas knackmussii* strain B13 and *Rhodococcus opacus* 1CP and potential impact on biomarker stable isotope probing. Environ Microbiol 10: 1641–1651. 18341583 10.1111/j.1462-2920.2008.01573.x

[3] Fenchel T, Blackburn TH (1979) Bacteria and mineral cycling. Academic Press, London.

[4] Southward AJ, Southward EC, Dando PR, Rau GH, Felbeck H, et al (1981) Bacterial symbionts and low 13C/12C ratios in tissues of Pogonophora indicate unusual nutrition and metabolism. Nature 293: 616–620.

[5] Levin LA, Mendoza GF, Konotchick T, Lee RW (2009) Macrobenthos community structure and trophic relationships within active and inactive Pacific hydrothermal sediments. Deep-Sea Research II 56: 1632–1648.

[6] Bastviken D, Ejlertsson J, Sundh I, Tranvik L (2003) Methane as a source of carbon and energy for lake pelagic food webs. Ecology 84: 969–981.

[7] Sanseverino AM, Bastviken D, Sundh I, Pickova J, Enrich-Prast A (2012) Methane Carbon Supports Aquatic Food Webs to the Fish Level. PLoS ONE 7(8): e42723. 22880091 10.1371/journal.pone.0042723 PMC3413669

[8] Garcia-Cantizano J, Casamayor EO, Gasol JM, Guerrero R, Pedros-Alio C (2005) Partitioning of CO_2_ incorporation among planktonic microbial guilds and estimation of in situ specific growth rates. Microb Ecol 50: 230–241. 16184336 10.1007/s00248-004-0144-9

[9] Detmer AE, Giesenhagen HC, Trenkel VM, Venne HAD, Jochem FJ (1993) Phototrophic and Heterotrophic Pico-Plankton and Nanoplankton in Anoxic Depths of the Central Baltic Sea. Mar Ecol: Prog Ser 99: 197–203.

[10] Jorgensen BB, Fossing H, Wirsen CO, Jannasch HW (1991) Sulfide Oxidation in the Anoxic Black-Sea Chemocline. Deep-Sea Res, Part A 38: 1083–1103.

[11] Taylor GT, Iabichella M, Ho TY, Scranton MI, Thunell RC, et al (2001) Chemoautotrophy in the redox transition zone of the Cariaco Basin: A significant midwater source of organic carbon production. Limnol Oceanogr 46: 148–163.

[12] Pedros-alio C, Guerrero R (1991) Abundance and activity of bacterioplankton in warm lakes. Verh Int Verein Limnol 24: 1212–1219.

[13] Kuuppo-Leinikki P, Salonen K (1992) Bacterioplankton in a small polyhumic lake with an anoxic hypolimnion. Hydrobiologia 222: 159–168.

[14] Camacho A, Erez J, Chicote A, Florin M, Squires MM, et al (2001) Microbial microstratification, inorganic carbon photoassimilation and dark carbon fixation at the chemocline of the meromictic Lake Cadagno (Switzerland) and its relevance to the foodweb. Aquat Sci 63: 91–106.

[15] Hadas O, Pinkas R (2001) High chemoautotrophic primary production in Lake Kinneret, Israel: A neglected link in the carbon cycle of the lake. Limnol Oceanogr 46(8): 1968–1976.

[16] Enrich-Prast A, Bastviken D, Crill PM (2009) Chemosynthesis. In: Likens G, editor. Encyclopedia of Inland Waters, Oxford, Elsevier, 211–225.

[17] Dean WE (1999) The carbon cycle and biogeochemical dynamics in lake sediments. J of Paleolimnol 21: 375–393.

[18] Heinen EA, McManus J (2004) Carbon and nutrient cycling at the sediment-water boundary in western Lake Superior. J Great Lakes Res 30: 113–132.

[19] Shively JM, van Keulen G, Meijer WG (1998) Something from almost nothing: Carbon dioxide fixation in chemoautotrophs. Annu Rev Microbiol 52: 191–230. 9891798 10.1146/annurev.micro.52.1.191

[20] Pimenov NV, Kallistova HYu, Rusanov II, Yusupov SK, Montonen L, et al (2010) Methane formation and oxidation in the meromectic oligotrophic Lake Gek-Gel (Azerbaijan). Microbiol 79(2): 247–252.

[21] Trimmer M, Grey J, Heppell CM, Hildrew AG, Lansdown K, et al (2012) River bed carbon and nitrogen cycling: State of play and some new directions. Sci Total Environ 434: 143–158. 22682557 10.1016/j.scitotenv.2011.10.074

[22] Grey JA, Kelly S, Ward N, Sommerwer K, Jones RI (2004) Seasonal changes in the stable isotope values of lakedwelling chironomid larvae in relation to feeding and life cycle variability. Freshwater Biol 49: 681–689.

[23] Kankaala P, Taipale S, Grey J, Sonninnen E, Arvola R, et al (2006) Experimental σ13C evidence for a contribution of methane to pelagic food webs in lakes. Limnol Oceanogr 51(6): 2821–2827.

[24] Rawcliffe R, Sayer CD, Woodward G, Grey J, Davidson TA, et al (2010) Back to the future: using palaeolimnology to infer long-term changes in shallow lake food webs. Freshwater Biol 55: 600–613.

[25] Ravinet M, Syväranta J, Jones RI, Grey J (2010) A trophic pathway from biogenic methane supports fish biomass in a temperate lake ecosystem. Oikos 119: 409–416.

[26] Schallenberg M, Kalff J (1993) The Ecology of Sediment Bacteria in Lakes and Comparisons with Other Aquatic Ecosystems. Ecology 74: 919–934.

[27] Sander BC, Kalff J (1993) Factors controlling bacterial production in marine and freshwater sediments. Microb Ecol 26: 79–99. 24190006 10.1007/BF00177045

[28] Gudasz C, Bastviken D, Steger K, Premke K, Sobek S, et al (2010) Temperature-controlled organic carbon mineralization in lake sediments. Nature 466: 478–481. 20651689 10.1038/nature09186

[29] Gudasz C, Bastviken D, Premke K, Steger K, Tranvik LJ (2012) Constrained microbial processing of allochthonous organic carbon in boreal lake sediments. Limnol Oceanogr 57(1): 163–175.

[30] Marotta H, Paiva LT, Petrucio MM (2009) Changes in thermal and oxygen stratification pattern coupled to persistence of CO2 outgassing in shallow lakes of the surroundings of Atlantic Tropical Forest, Brazil. Limnol 10: 195–202.

[31] Richey JE, Melack JM, Aufdenkampe AK, Ballester VM, Hess LL (2002) Outgassing from Amazonian rivers and wetlands as a large tropical source of atmospheric CO2. Nature 416: 617–620. 11948346 10.1038/416617a

[32] Brown JH, Gillooly JF, Allen AP, Savage VanM, West GB (2004) Toward a metabolic theory of ecology. Ecology 85: 1771–1789.

[33] Luyssaert S, Inglima I, Jungs M, Richardson AD, Reichsteins M, et al (2007) CO_2_ balance of boreal, temperate, and tropical forests derived from a global database. Global Change Biol 13: 2509–2537.

[34] Dalsgaard R, Nielsen LP, Brotas V, Viaroli P, Underwood G, et al.. (2000) Protocol handbook for NICE – Nitrogen Cycling in Estuaries: a project under the EU research programme: Marine Science and Technology (MAST III). National Environmental Research Institute, Silkeborg, Denamark. 62p.

[35] Haglund AL, Lantz P, To”rnblom E, Tranvik L (2003) Depth distribution of active bacteria and bacterial activity in lake sediment. FEMS Microbiol Ecol 46: 31–38. 19719580 10.1016/S0168-6496(03)00190-9

[36] Buesing N, Marxsen J (2005) Theoretical and empirical conversion factors for determining bacterial production in freshwater sediments via leucine incorporation (vol 3, pg 101, 2005). Limnol Oceanog: Methods 3: 221–221.

[37] Sweerts J, Bargilissen MJ, Cornelese AA, Cappenberg TE (1991) Oxygen-Consuming Processes at the Profundal and Littoral Sediment Water Interface of a Small Meso-Eutrophic Lake (Lake Vechten, the Netherlands). Limnol Oceanogr 36: 1124–1133.

[38] Tomaszek JA, Czerwieniec E (2003) Dentrification and oxygen consumption in bottom sediments: factors influencing rates of the processes. Hydrobiologia 504: 59–65.

[39] Christian BW, Lind OT (2007) Increased sediment-water interface bacterial [H-3]-L-serine uptake and biomass production in a eutrophic reservoir during summer stratification. Fundam Appl Limnol 168: 189–199.

[40] Evrard V, Cook PLM, Veuger B, Huettel M, Middelburg JJ (2008) Tracing carbon and nitrogen incorporation and pathways in the microbial community of a photic subtidal sand. Aquat Microb Ecol 53: 257–269.

[41] Thomsen U, Kristensen E (1997) Dynamics of Sigma CO2 in a surficial sandy marine sediment: The role of chemoautotrophy. Aquat Microb Ecol 12: 165–176.

[42] Mitsch WJ, Gosselink JG (2007) Wetlands, 4th ed. Wiley, NewYork.

[43] Galchenco VF (1994) Sulfate reduction, methane production, and methane oxidation in various water bodies of Banger-Hills Oasis of Antarctica. Microbiol 63(4): 388–396.

[44] Scheffer M (1998). Ecology of Shallow Lakes. Chapman & Hall, London.

[45] Kranzberg G (1985) The influence of bioturbation on physical and biological parameters in aquatic environments: a review. Environ Pollut, Ser A 39: 23.

[46] Svensson JM, Enrich-Prast A, Leonardson L (2001) Nitrification and denitrification in a eutrophic lake sediment bioturbated by oligochaetes. Aquat Microb Ecol 23: 177–186.

[47] Heip CHR, Goosen NK, Herman PMJ, Kromkamp J, Middelburg JJ, et al (1995) Production and consumption of biological particles in temperate tidal estuaries. Oceanogr Mar Biol Annu Rev 33: 1–149.

[48] Molari M, Manini E, Dell’Anno A (2013) Dark inorganic carbon fixation sustains the functioning of benthic deep-sea ecosystems. Global Biogeochem Cy 27: 1–10.

[49] Thauer RK (1988) Citric-Acid Cycle, 50 Years on - Modifications and an Alternative Pathway in Anaerobic-Bacteria. Eur J Biochem 176: 497–508. 3049083 10.1111/j.1432-1033.1988.tb14307.x

[50] Erb TJ, Brecht V, Fuchs G, Muller M, Alber BE (2009) Carboxylation mechanism and stereochemistry of crotonyl-CoA carboxylase/reductase, a carboxylating enoyl-thioester reductase. Proc Natl Acad Sci U. S. A. 106: 8871–8876. 10.1073/pnas.0903939106 PMC2689996 19458256

[51] Schink B (2009) An alternative to the glyoxylate shunt. Mol Microbiol 73: 975–977. 19682245 10.1111/j.1365-2958.2009.06835.x

[52] Merlin C, Masters M, McAteer S, Coulson A (2003) Why is carbonic anhydrase essential to Escherichia coli? J Bacteriol 185: 6415–6424. 14563877 10.1128/JB.185.21.6415-6424.2003 PMC219403

[53] Haglund AL, Tornblom E, Bostrom B, Tranvik L (2002) Large differences in the fraction of active bacteria in plankton, sediments, and biofilm. Microb Ecol 43: 232–241. 12023730 10.1007/s00248-002-2005-0

[54] Alonso-Saez L, Galand PE, Casamayor EO, Pedros-Alio C, Bertilsson S (2010) High bicarbonate assimilation in the dark by Arctic bacteria. ISME J 4: 1581–1590. 20555365 10.1038/ismej.2010.69

[55] Fenchel T (2008) The microbial loop –25 years later. J Exp Mar Biol Ecol. 366: 99–103.

